# Prosthetic joint infection caused by *Granulicatella adiacens*: a case series and review of literature

**DOI:** 10.1186/s12891-017-1630-1

**Published:** 2017-06-23

**Authors:** Fanny Quénard, Piseth Seng, Jean-Christophe Lagier, Florence Fenollar, Andreas Stein

**Affiliations:** 1 Centre de Référence des Infections Ostéo-Articulaires (CRIOA) Sud-Méditerranée, Assistance Publique - Hôpitaux de Marseille, IHU–Méditerranée Infection, 19–21 Boulevard Jean Moulin, 13005 Marseille, France; 2 Service de Maladies Infectieuses Tropicales et Infections Chroniques (MITIC), IHU–Méditerranée Infection, 19–21 Boulevard Jean Moulin, 13005 Marseille, France; 3 Aix-Marseille Université, Unité de recherche sur les maladies infectieuses et tropicales émergentes (URMITE), UM63, CNRS 7278, IRD 198, INSERM 1095, IHU–Méditerranée Infection, 19-21 Boulevard Jean Moulin, 13005 Marseille, France

**Keywords:** Prosthetic joint infection, Arthroplasty, *Granulicatella adiacens*, Nutritionally variant streptococcus, Osteoarthritis, Arthritis, Infection, Bacteria, Human

## Abstract

**Background:**

Bone and joint infection involving *Granulicatella adiacens* is rare, and mainly involved in cases of bacteremia and infectious endocarditis. Here we report three cases of prosthetic joint infection involving *G. adiacens* that were successfully treated with surgery and prolonged antimicrobial treatment. We also review the two cases of prosthetic joint infection involving *G. adiacens* that are reported in the literature.

**Case presentation:**

Not all five cases of prosthetic joint infection caused by *G. adiacens* were associated with bacteremia or infectious endocarditis. Dental care before the onset of infection was observed in two cases. The median time delay between arthroplasty implantation and the onset of infection was of 4 years (ranging between 2 and 10 years). One of our cases was identified with 16srRNA gene sequencing, one case with MALDI-TOF mass spectrometry, and one case with both techniques. Two literature cases were diagnosed by 16srRNA gene sequencing. All five cases were cured after surgery including a two-stage prosthesis exchange in three cases, a one-stage prosthesis exchange in one case, and debridement, antibiotics, irrigation, and retention of the prosthesis in one case, and prolonged antimicrobial treatment.

**Conclusion:**

Prosthetic joint infection involving *G. adiacens* is probably often dismissed due to difficult culture or misdiagnosis, in particular in the cases of polymicrobial infection. Debridement, antibiotics, irrigation, and retention of the prosthesis associated with prolonged antimicrobial treatment (≥ 8 weeks) should be considered as a treatment strategy for prosthetic joint infection involving *G. adiacens*.

## Background

*Granulicatella adiacens* is a nutritionally variant streptococci that is known as a commensal human mouth flora [1]. *Granulicatella adiacens* is usually involved in cases of bacterial endocarditis [2–5] and bacteremia [6, 7]. Bone and joint infection involving *G. adiacens* is rare. In this study, we report three cases of prosthetic joint infection caused by *G. adiacens* treated in our center for bone and joint infection. We also reviewed literature cases of prosthetic joint infection involving *G. adiacens* (Table 1).

**Table 1:** Clinical characteristics, treatment strategies and outcomes of the five cases of prosthetic joint infection caused by Granulicatella adiacens

Number of cases	Age (years)	Sex	Location of Infection	Time delay between arthroplasty implantation and infection onset	Dental care before infection onset	Microbiologic diagnostics of *G. adiacens*	Associated microorganisms	Surgery treatment options	Antibiotics	Outcomes
Our 1st case	75	Male	Hip arthroplasty	4 years	Yes	Microbial culture of surgical biopsies was negative.	*Parvimonas micra*	Two-stage prosthesis exchange	Amoxicillin and clindamycin	Cured
						16S rRNA gene sequencing on synovial fluid was positive.				
Our 2nd case	65	Male	Knee arthroplasty	2 years	No	Maldi-Tof mass spectrometry on bacterial colonies.	*Staphylococcus capitis*	One-stage prosthesis exchange	Rifampicin and clindamycin	Cured
Our 3rd case	44	Female	Hip arthroplasty	10 years	No	Maldi-Tof mass spectrometry on bacterial colonies	*Klebsiella pneumoniae*	Debridement, antibiotics, irrigation, and retention of the prosthesis (DAIR)	Imipenem-cisplatin then ciprofloxacin and amoxicillin	Cured
						16S rRNA gene sequencing on synovial fluid was positive.				
Riede et al., 2004 [12]	43	Male	Knee arthroplasty	3 years	No	Microbial culture of surgical biopsies was positive but the microorganism could not be identified reliably by phenotypic methods	No	Two-stage prosthesis exchange	Amoxicillin, amikacin and rifampicin	Cured
						16S rRNA gene sequencing on bacterial colonies				
Mougari et al., 2013 [13]	55	Male	Knee arthroplasty	10 years	Yes	Microbial culture of surgical biopsies was negative.	No	Two-stage prosthesis exchange	Amoxicillin and rifampicin	Cured
						16S rRNA gene sequencing on synovial fluid was positive.				

## Case presentation

### Case 1

In June 2013, a 75-year-old French male was admitted to our center for a fistula and purulent discharge from the scar of a hip prosthesis. His medical history included high blood pressure, ankylosing spondylitis and sleep apnea. In 2009, he underwent a left hip prosthesis procedure for a femoral head avascular necrosis. In 2012, he was admitted to a private hospital for reddish, painful hip prosthesis. He presented a fistula and purulent discharge from the hip prosthesis surgical scar. He denied fever. He underwent a dental extraction three months earlier. Microbial cultures of purulent discharge were for methicillin-susceptible *S. aureus*. He was treated with three months of oral ciprofloxacin, 500 mg three times daily, and oral fusidic acid, 500 mg, three times daily. At the end of the antimicrobial treatment, he presented a persistent fistula and purulent discharge from the hip prosthesis surgical scar. He was treated with prosthetic debridement, antibiotics, irrigation, and retention (DAIR) and one year of antibiotic treatment with oral rifampicin, 300 mg, three times daily, and oral ofloxacin, 200 mg, three times daily. Microbial cultures of surgical biopsies were negative.

When he arrived, laboratory investigations revealed a normal value for C-reactive protein (5 mg/L) and a normal leukocyte count (6000 μL^−1^). He was treated with two-stage exchange arthroplasty. Microbial cultures of surgical biopsies were positive for *Parvimonas micra* as identified by MALDI-TOF mass spectrometry on colonies which have grown in blood culture bottle containing synovial fluid. 16S rRNA gene sequencing directly on synovial fluid was positive for *G. adiacens*. He was treated with 6 months of oral amoxicillin, 2 g, three times daily, and oral clindamycin, 9 g, three times daily. Clinical outcome post-prosthesis removal was good with the disappearance of the fistula, but he presented posterior luxation of the hip spacer. A new hip prosthesis was implanted 3-months post-removal. No relapse was observed during the two-year post-antimicrobial follow-up consultation.

### Case 2

In January 2014, a 65-year-old French male was admitted to our center for knee prosthesis loosening. In 2002, he presented destructive arthritis treated with implantation of a unicompartmental left knee arthroplasty. His medical history included psoriasis, chronic alcoholism and oesophagitis. In October 2011, he presented left knee prosthesis loosening and underwent replacement of a unicompartmental knee arthroplasty by a total knee prosthesis. Two months later, he presented a prosthetic join infection with surgical biopsies positive for *S. aureus* and *S. epidermidis*. He was initially treated with debridement, antibiotics, irrigation, and retention of prosthetic (DAIR) followed by a two-stage exchange arthroplasty and 8 months of oral rifampicin, 300 mg, three times daily, and ofloxacin, 200 mg, three times daily. In November 2012, he fell and presented persistent left knee prosthesis pain without any abnormality in the knee X-ray. In January 2014, he arrived in our center for knee prosthetic loosening. Laboratory investigations revealed a high value for C-reactive protein (28 mg/L; normal values ≤5 mg/L) and a leukocyte count of 8400 μL^−1^. He was treated with one-stage revision of knee prosthesis. Blood culture bottle containing synovial fluid, after incubation, was positive for *G. adiacens* and *S. capitis* using MALDI-TOF identification on bacterial colonies*.* He was treated with 6 months of oral rifampicin, 300 mg, three times daily, and oral clindamycin, 9 g, three times daily. No relapse was observed during the two-year post-antimicrobial follow-up.

### Case 3

In April 2015, a 44-year-old French female was admitted to our center with a one-year history of periprosthetic cyst formation associated with joint pain and a surgical scar fistula. Ten years earlier, she underwent a bilateral hip prosthesis implantation for congenital hip dysplasia. A cystic lesion appeared around the left hip joint two years before her admission. For that lesion, she underwent a surgical resection of the cystic lesion around the hip joint. Microbiological cultures of surgical deep samples were negative. Upon admission, she presented no fever but left hip joint pain and a fistula with purulent discharge from the surgical wound. Laboratory tests revealed a high leukocyte count of 11,000 μL^−1^ and a normal value for C-reactive protein of 5 mg/L. The hip radiograph showed no evidence of hip arthroplasty loosening. Bacterial cultures of surgical deep samples were positive for *G. adiacens* and *Klebsiella pneumonia,* as identified using MALDI-TOF identification on bacterial colonies. In parallel, 16S rRNA gene sequencing directly on synovial fluid was positive for *G. adiacens*. She was treated with debridement, irrigation with implant retention (DAIR) and antimicrobial treatment with one month of intravenous imipenem/cilastatin, 1 g, twice daily, and oral ciprofloxacin, 500 mg, three times daily, followed by 5 months of oral amoxicillin, 2 g, three times daily, and oral ciprofloxacin 500 mg, three times daily. No relapse was observed during the 16-month post-antimicrobial follow-up.

## Discussion

Bone and joint infection caused by *G. adiacens* is rarely reported. To the best of knowledge, eight cases have been published to date, including five cases of vertebral osteomyelitis [2, 8–10], one case of native arthritis [11] and two cases of prosthetic joint infection [12, 13]. Here we report three cases of prosthetic joint infection caused by *G. adiacens* treated in our center. We believe that this organism may be still under-reported as a pathogen in prosthetic joint infection.

Cases of *G. adiacens* infection may be difficult to diagnose due to their slow growth characteristics. The microorganism is sometimes dismissed by biochemical testing and often needs confirmation by molecular techniques [7]. In our first case, *G. adiacens* infection was identified at the end of the antimicrobial treatment for *S. aureus* PJI. We believe that the patient was initially infected with both *S. aureus, Parvimonas micra* and *G.adiacens.* These two last pathogens were probably misidentified on previous surgical biopsies by classical culture and have been identified only after the optimal treatment for *S. aureus* infection with a combination of ofloxacin and rifampicin. Recently, MALDI-TOF mass spectrometry has been reported to be a rapid and accurate tool for identifying *G. adiacens* [14]. Application in clinical laboratories of MALDI-TOF mass spectrometry has revolutionized routine bacterial identification that have become more rapid, accurate and less expensive [15]. We believe that the availability of these molecular identification techniques or MALDI-TOF mass spectrometry will help clinicians in increasing the number of diagnosis of *G. adiacens* infection cases.In our center, the protocol for the diagnosis of prosthetic joint infection contains surgical biopsies obtained from all patients i.e., joint fluid, bone biopsies or tissue samples around joint prosthesis, which were crushed in Eppendorf (Hamburg, Germany) tubes and inoculated on 5% sheep-blood, chocolate, Mueller-Hinton, trypticase soy and MacConkey agar plates (BioMérieux, France) and incubated at 37 °C in a 5% CO2 atmosphere and in an anaerobic atmosphere for 15 days. Pure bacterial cultures, obtained by picking isolated colonies, were identified with conventional phenotypic identification methods such as Gram staining (Aerospray Wiescor; Elitech), catalase and oxidase activity tests, automated phenotypic identification systems including the Vitek 2 system (BioMérieux, Marcy l’Etoile, France), MALDI-TOF mass spectrometry or molecular methods, as previously described [16]. One of our case and one other case in literature [13] had negative microbial culture of surgical biopsies or synovial fluid. Two of our cases and one case in literature [13] were identified with 16srRNA gene sequencing on synovial fluid. Two of our cases were identified with MALDI-TOF mass spectrometry on bacterial colonies grown from cultures of surgical biopsies.

*G. adiacens* is a commensal bacteria and part of the oral flora. This localization may play a role in the potential bloodstream infection in patients with a history of oral care or subcutaneous dissemination of prosthetic joint infection, which usually involves another microorganism colonizing the oral cavity or the skin. Significant role of *G. adiacens* in polymicrobial prosthetic joint infection should be considered if the organism is isolated from ≥2 per-operative surgical biopsies. The median time delay between arthroplasty implantation and the onset of infection for the five cases of prosthetic joint infection caused by *G. adiacens* was of 4 years (ranging between 2 and 10 years). This might be explained by the fact that this organism comes from the hematogenous infection of the oral cavity. However, not all of the five cases of prosthetic joint infection caused by *G. adiacens* were associated with bacteremia or infectious endocarditis; and dental care before the onset of infection was observed in only two cases including one case in our study and in one case in the literature [13]. Two of our cases (Case 1 and Case 2) and one case reported by Riede et al. were diagnosed after antimicrobial treatments for prosthetic joint infection caused by staphylococci and other pathogens. Diagnosis of *G. adiacens* infection should be investigated by using modern microbial identification techniques such as MALDI-TOF mass spectrometry or molecular tools when general antimicrobial treatment for prosthetic joint infection has failed.

All the cases of prosthetic joint infection caused by *G. adiacens* were treated by surgery including a two-stage prosthesis exchange in three cases, a one-stage prosthesis exchange in one case, and debridement, antibiotics, irrigation, and retention of the prosthesis (DAIR) in one case, followed by a prolonged antimicrobial treatment (≥ 8 weeks). All of our cases were treated with 6 months of antimicrobial treatment. The duration of antimicrobial treatment in our three cases (180 days) was longer than for the cases reported in the literature (56 to 104 days); no relapse was observed in our cases or in the cases reported in the literature. An increased number of studies on prosthetic joint infection caused by *G. adiacens* is needed to clarify treatment strategies, including duration of antimicrobial treatment and surgical treatment options. One of our cases was cured with debridement antibiotics, irrigation, and retention of the prosthesis (DAIR) associated with prolonged antimicrobial treatment. However, more data are needed to confirm that DAIR and prolonged antimicrobial treatment (≥ 8 weeks) can be sufficient in the treatment of prosthetic joint infection caused by *G. adiacens.*

## Conclusion

*G. adiacens* is a virulent pathogen in prosthetic joint infection. Cases of prosthetic joint infections due to *G. adiacens* are probably often dismissed due to difficult culture or misdiagnosis, and particularly in the case of polymicrobial infection. Prolonged cultures of surgical biopsies and the choice of optimal identification techniques such as molecular tools or MALDI-TOF mass spectrometry can help clinicians to diagnose these cases. Surgery and prolonged antimicrobial treatment (≥ 8 weeks) were needed to control infection.

## Abbreviations

DAIR: Debridement, antibiotics, irrigation, and retention
MALDI-TOF mass spectrometry: The matrix-assisted laser desorption/ionization-time of flight mass spectrometry
